# Utility of Expandable Interbody Cages in Open Transforaminal Interbody Fusions: A Comparison With Static Cages

**DOI:** 10.7759/cureus.40262

**Published:** 2023-06-11

**Authors:** Alexander G Yearley, Joshua I Chalif, Hasan A Zaidi

**Affiliations:** 1 Department of Neurological Surgery, Harvard Medical School, Boston, USA; 2 Department of Neurological Surgery, Brigham and Women's Hospital, Boston, USA

**Keywords:** spinal deformity, adult spinal deformity, transforaminal lumbar interbody fusion, transforaminal lumbar interbody fusion (tlif), open surgery, spine surgery, pedicle subtraction osteotomy, expandable cage, adult spine deformity

## Abstract

Background

Expandable interbody cages, while popular in minimally invasive fusions due to their slim profile and increased ease of insertion, have not been widely explored in open surgery. The benefits of expandable cages may also extend to open fusions through their potential to achieve a greater restoration of lumbar lordosis while minimizing intraoperative complications. To highlight these benefits, we present a case series of adult spinal deformity (ASD) patients treated with an open transforaminal lumbar interbody fusion (TLIF) using expandable cages and compare outcomes to those of patients treated with static cages from the literature.

Methods

A retrospective cohort study of patients who underwent a deformity correction procedure and TLIF with expandable interbody cages at Brigham and Women's Hospital between 2018 and 2022 was conducted. Patient demographics, complications, and pre- and postoperative radiographic parameters of spinopelvic alignment were collected. A literature search was completed to identify studies employing static cages. T-tests were performed to compare postoperative changes in radiographic parameters by cage type.

Results

Forty-five patients (mean age of 62.6 years) with an average of 2.1 cages placed met the inclusion criteria. Patients experienced five intraoperative complications and 23 neurologic deficits (from minor to major), while nine patients required a revision operation. Lumbar lordosis increased by 9.8° ± 14.5° (p < 0.0001), the sagittal vertical axis (SVA) decreased by 25.5 mm ± 56.7 mm (p = 0.0048), and pelvic incidence-lumbar lordosis mismatch decreased by 13.3° ± 17.5° (p < 0.0001) with the use of expandable cages. Expandable cages yielded similar changes in lumbar lordosis to 15° and 8° cages but improved the lumbar lordosis generated from rectangular and 4° cages. When compared to static cages, expandable cages mildly reduced intraoperative complications.

Conclusions

Expandable interbody cages are an effective means of restoring spinopelvic alignment in ASD that have the potential to improve patient outcomes in open fusions compared to standard static cages. Especially when compared to rectangular and 4° static cages, expandable cages provide a clear benefit in the correction of lumbar lordosis. The impact of open spinal fusions with expandable cages on outcomes should continue to be explored in other cohorts.

## Introduction

Adult spinal deformity (ASD) is a progressive degenerative condition of the thoracolumbar spine most commonly seen in aging populations. By some estimates, the prevalence of ASD has as much as tripled in recent years, with further increases expected as the world population continues to age [1]. If left untreated, spine deformity is associated with significant morbidity and a decreased quality of life [2,3]. With multiple treatment options available, the optimal management strategy is tailored to the individual’s level of pain, deformity, and disability [4]. While surgery is considered a last resort, multiple techniques and approaches are available.

Open transforaminal lumbar interbody fusion (TLIF) is a commonly employed procedure in ASD surgery. The TLIF only involves a unilateral facetectomy, which limits spinal trauma, reduces damage to ligamentous structures, and minimizes nerve injury compared to the formerly employed posterior lumbar interbody fusion [5]. However, TLIFs have been criticized as providing insufficient correction of lumbar lordosis and coronal imbalance [6]. Variation in the type of interbody cage used offers one solution to this problem. TLIFs with lordotic cages have been associated with an increased restoration of lumbar lordosis compared to flat, rectangular cages [7,8].

Expandable interbody cages were developed as an alternative to static cages, which, due to their larger size, have been linked to an increased risk of dural laceration and nerve root injury during placement [9]. After proper positioning, the collapsed cage can be expanded to a height determined by the operator. The reduced profile and increased control associated with the use of the expandable cage have made it popular in minimally invasive (MIS) lumbar interbody fusions [10]. Multiple studies have shown that expandable cages improve disc height and lumbar lordosis when employed in MIS TLIFs [11,12]. However, the role of expandable cages in open spinal fusions remains poorly defined. There is a utility for expandable cages in adult deformity. By increasing anterior column length and supplementing with posterior osteotomies, a greater restoration of lumbar lordosis can be achieved. The authors believe that these benefits are not limited to MIS surgery and that the use of expandable cages should be considered in open TLIFs. To investigate these potential benefits, we present an institutional case series of spine deformity patients treated with open TLIFs with the placement of expandable interbody cages. Complications and radiographic parameters of spinopelvic alignment are reported and compared to those of static interbody cages from the literature to determine the true benefit of expandable cages in open surgery.

## Materials and methods

Study sample

A retrospective chart review of 114 patients who underwent an open TLIF at Brigham and Women's Hospital between 2018 and 2022 was conducted. This study was performed using a de-identified patient database and was exempt from Institutional Review Board approval.

Inclusion criteria

To meet inclusion criteria, patients must have had at least one expandable interbody cage (Depuy Concorde Lift, Nuvasive TLX Cage, Medtronic Catalyft) placed during fusion and complete pre- as well as postoperative imaging within a year of the index operation, respectively. Indications for surgery included thoracolumbar scoliosis, spinal stenosis, hardware failure, chronic pain, and degenerative disc disease. Both primary and revision operations were included in this analysis.

Exclusion criteria

Patients with indications related to infection, malignancy, or trauma were excepted from this analysis. Patients treated with MIS, lateral, or anterior approaches were excluded. Any patient who underwent a higher-grade osteotomy (>Schwab grade 2) was also excluded.

Clinical & demographic variables

Demographic data were notated for each patient, including age at operation, sex, body mass index (BMI), number of comorbidities, smoking status, and pre-operative American Society of Anesthesiologists (ASA) grade. Surgery type, number of levels instrumented, number of cages employed, blood loss, operative time defined as the time from first incision to closure, length of hospitalization, and length of intensive care unit (ICU) stay were collected for each procedure.

Intraoperative, postoperative, and post-discharge complications were collected for each patient. Intraoperative complications were defined as intraoperative neurologic deficits, cerebrospinal fluid (CSF) leak, pedicle or vertebral body fracture, inadvertent extubation, or death. Post-discharge, patients were followed for hardware failure, proximal junctional kyphosis, and the need for a revision operation.

Radiographic evaluation

Pre- and postoperative standing anteroposterior (AP) and lateral X-ray scoliosis films were available for all included patients. Films acquired closer to the index operation were preferred, but any imaging acquired within a year of surgery was included. If postoperative scoliosis radiographs occurred after a second revision operation, they were excluded. Maximum thoracolumbar Cobb angle was measured from AP films. Lumbar lordosis, thoracic kyphosis, sagittal imbalance (i.e., sagittal vertical axis (SVA)), pelvic incidence, and pelvic tilt were derived from lateral radiographs. Standard sign conventions were employed in the measurement of SVA with positive values representing a C7 plumb line anterior to the central line and negative values representing a C7 plumb line posterior to the central line.

Literature search

A search strategy was implemented in PubMed using keywords related to “fusion,” “cage,” and “spine deformity.” Abstracts and full texts were screened to identify studies utilizing static cages in open spinal fusions with posterior or transforaminal approaches for ASD. Included studies were extracted for cohort demographics, complications, revision rates, and pre- as well as postoperative radiographic parameters of spinopelvic alignment.

Statistical analysis

When available, variable distributions were presented as the mean ± standard deviation (SD). Paired two-tailed t-tests were employed to compare pre- and postoperative radiographic parameters from the same study cohort. Independent two-tailed t-tests were utilized for all other comparisons of both clinical and radiographic variables between cohorts. For a minority of studies identified in the literature search, pre- and postintervention SDs were available without the reporting of the change-from-baseline SD for radiographic parameters. For such studies where raw data were not available, an alternative approach was considered to approximate the SD of the change-from-baseline. In line with Cochrane Handbook for Systematic Reviews of Interventions guidelines, change from baseline SDs were computed according to the following formula [13]:

$SD_{E,change}=\sqrt{SD_{E,baseline}^2 + SD_{E,final}^2-\left ({2\times Corr\times SD_{E,baseline} \times SD_{E,final}}\right )}$

The correlation coefficient (Corr) was derived from the corresponding radiographic parameter in the expandable cage cohort. For example, for studies missing the SD of the change in lumbar lordosis, the calculated correlation coefficient for the SD of the change in lumbar lordosis in the expandable cage cohort was substituted.

Significance was set as a p-value ≤ 0.05. All statistical analysis was conducted in MATLAB version 9.12.0 (MathWorks, Natick, Massachusetts).

## Results

Cohort

Of the 114 ASD patients screened, 45 met the study's inclusion criteria with a mean age of 62.6 (SD = 8.2) years at index operation (Table 1). The cohort was 62.2% female, had a mean of 3.7 (SD = 2.4) comorbidities, and a mean ASA grade of 2.8 (SD = 0.5). All 45 patients underwent an open TLIF with the placement of a mean of 2.1 (SD = 0.9) expandable cages. Expandable cages were most frequently placed at levels L3-L4, L4-L5, and L5-S1 (20.0%); L4-L5 and L5-S1 (20.0%); and at L5-S1 (22.2%) alone. A mean of 8.4 (SD = 3.5) levels was instrumented with 24.4% of cases being a revision operation. Not a single patient received an osteotomy during their procedure. Patients had a median of 1.5 (range = 0.5-4.0) years of follow-up.

**Table 1:** Demographics and surgical data of the study cohort. SD, standard deviation; BMI, body mass index; ASA, American Society of Anesthesiologists; ICU, intensive care unit.

Variable	Value (mean ± SD, ratio, n (%))
Age at operation (years)	62.6 ± 8.2
Male to female ratio	17:28
BMI (kg/m^2^)	28.8 ± 5.7
No. of comorbidities	3.7 ± 2.4
Current smokers	4 (8.9%)
ASA grade	2.8 ± 0.5
Revision cases	11 (24.4%)
No. of levels instrumented	8.4 ± 3.5
T2-ilium	2 (4.4%)
T3-ilium	1 (2.2%)
T4-ilium	5 (11.1%)
T5-ilium	1 (2.2%)
T9-L3	1 (2.2%)
T9-ilium	4 (8.9%)
T10-ilium	18 (40.0%)
T11-ilium	2 (4.4%)
T12-ilium	1 (2.2%)
L2-ilium	10 (22.2%)
No. of cages placed	2.1 ± 0.9
T11-12, T12-L1	1 (2.2%)
L1-L2, L2-L3	1 (2.2%)
L1-L2, L4-L5	1 (2.2%)
L2-L3	1 (2.2%)
L2-L3, L3-L4	2 (4.4%)
L2-L3, L3-L4, L4-L5	1 (2.2%)
L2-L3, L3-L4, L4-L5, L5-S1	3 (6.7%)
L2-L3, L3-L4, L5-S1	2 (4.4%)
L2-L3, L5-S1	1 (2.2%)
L3-L4, L4-L5, L5-S1	9 (20.0%)
L3-L4, L5-S1	1 (2.2%)
L4-L5	3 (6.7%)
L4-L5, L5-S1	9 (20.0%)
L5-S1	10 (22.2%)
Blood loss (mL)	2289 ± 2632
Operative time (min)	641.3 ± 112.7
Duration of hospital stay (days)	7.4 ± 4.7
Duration of ICU stay (days)	1.4 ± 4.0

There were five intraoperative complications including three CSF leaks, one pedicle infraction, and one transient cauda equina deficit, which resolved postoperatively. Sixteen patients experienced neurological deficits in the postoperative and follow-up periods. Postoperatively, there were four motor deficits, of which two had resolved at follow-up, two sensory deficits, both of which resolved, and three bladder deficits, of which one resolved at follow-up. Fourteen minor neurologic deficits occurred at follow-up, including three patients with numbness, eight with weakness, and three with both numbness and weakness. There were 10 instances of instrument failure, of which eight required a revision operation. One additional patient required revision for a vertebral body fracture.

Radiographic measures

Table 2 reports pre- and postoperative radiographic measurements of spine deformity for all 45 patients. Each radiographic measure significantly improved in the postoperative period (p ≤ 0.05). Maximum thoracolumbar Cobb angle decreased by a mean of 18.1° ± 12.2° (p < 0.0001). Lumbar lordosis significantly increased from 33.1° ± 17.2° pre-operatively to 42.9° ± 10.1° postoperatively (p < 0.0001). The degree of change in lumbar lordosis varied by patient with six patients experiencing an increase in lumbar lordosis by greater than 25° while 14 patients experienced a net decrease in lumbar lordosis. While pelvic incidence-lumbar lordosis (PI-LL) mismatch improved by 13.3° ± 17.5° (p < 0.0001), the postoperative PI-LL of 14.4° ± 10.9° failed to pass the 10° threshold that is associated with improved ASD outcomes [14-17]. Ultimately, 13 of 45 patients had a postoperative PI-LL of less than 10°. Expandable cages significantly and consistently improved the SVA to less than 50 mm, with an average postoperative SVA of 30.3 mm ± 43.0 mm in this cohort. Four patients experienced a decrease in SVA of greater than 100 mm.

**Table 2:** Pre- and postoperative radiographic measures from a cohort of spine deformity patients who underwent open transforaminal interbody fusion utilizing expandable cages. “Δ” represents the change in the radiographic measure after the index operation. PI-LL: pelvic incidence-lumbar lordosis.

Radiographic measure	Pre-Op	Post-Op	Δ	P
Thoracolumbar maximum Cobb angle (°)	30.1 ± 16.8	12.0 ± 8.2	−18.1 ± 12.2	<0.0001
Sagittal vertical axis (mm)	55.8 ± 45.6	30.3 ± 43.0	−25.5 ± 56.7	0.0048
Thoracic kyphosis (°)	35.7 ± 13.7	45.6 ± 11.8	9.8 ± 10.4	<0.0001
Lumbar lordosis (LL) (°)	33.1 ± 17.2	42.9 ± 10.1	9.8 ± 14.5	<0.0001
Pelvic incidence (PI) (°)	60.9 ± 12.0	57.4 ± 10.4	−3.5 ± 10.1	0.026
PI-LL mismatch (°)	27.8 ± 17.0	14.4 ± 10.9	−13.3 ± 17.5	<0.0001
Pelvic tilt (°)	26.6 ± 8.8	20.5 ± 8.6	−6.1 ± 8.2	<0.0001

## Discussion

One of the major goals of adult deformity surgery is to restore lumbar lordosis to help improve back pain and disability [18]. These procedures lack a “one size fits all” approach. For each patient, the surgeon must consider their approach, the use of interbody cages, and even the type of interbody cage to employ. Multiple interbody cages have been developed, including static rectangular, static bullet-shaped, static lordotic cages, expandable cages, and others, to restore disc height [7,19,20]. However, it is often challenging to place large TLIF cages that maximize anterior column lengthening without increasing the risk of nerve injury during placement. In this study, we investigated ­­­­the clinical and radiographic outcomes of patients treated with expandable interbody cages in open TLIFs. While commonly employed in MIS, this is the first case series to report on the use of expandable cages in open adult deformity surgery. Our personal experience has demonstrated improved restoration of lumbar lordosis with reduced nerve injury complications, as we typically employ these cages to maximize anterior column lengthening and supplement this with posterior column osteotomies.

The popularity of expandable cages in MIS is driven by the increased control and reduced profile associated with the collapsed cage [10]. MIS-TLIFs have been associated with lower complication rates, length of hospital stay, and blood loss [21]. While many of these benefits are conceivably due to the less invasive nature of the operation, this study sought to understand if some of the benefits came from the use of the expandable cage itself. In a meta-analysis of 79 studies of ASD surgery, Akıntürk et al. [22] found an average rate of neurologic complications of 10.8% (range: 0.3-35.5%). The rate of neurologic complications in this expandable cage cohort was 35.6%, which was within the range of expected neurologic complications. The expandable cage cohort had a 22.2% rate of instrument failure and a 20.0% revision rate, compared to the mean rate of instrument failure of 15.3% (0.7-62.5%) and the mean revision rate of 17.8% (10.3-53.7%) reported by Akıntürk et al. These results suggest that expandable cages yield clinical outcomes of instrument failure and revision rate that are nonsuperior to standard rates reported in the literature. However, expandable cages mildly reduced intraoperative complications overall. Multiple studies of ASD surgery have reported intraoperative complication rates of 14.0-17.4% [23,24]. The 11.1% intraoperative complication rate observed in this cohort was lower than that reported in the literature, perhaps due to the reduced profile of expandable cages during placement. In our experience, adult deformity patients with collapsed disc heights and a decreased size of Kambin’s triangle benefit from expandable cages because they allow for safer placement of larger cages without injuring the neural elements.

A literature search identified seven studies that evaluated the radiographic outcomes of patients after the placement of static interbody cages for ASD. The studies included a total of 503 patients with a mean age of 60.6 years. All cages were placed through an open posterior or transforaminal approach. Table 3 summarizes basic demographic information for each cohort. The included studies investigated three lordotic static cages, including those with 15° lordosis, 8° lordosis, and 4° lordosis, as well as static rectangular cages that lack lordosis.

**Table 3:** Study demographics and types of cages used for identified cohorts of patients undergoing posterior or transforaminal interbody fusion with static cages. When available, data are presented as mean ± standard deviation. † Two patients were treated with structural allograft instead of a rectangular cage.

Study	Cage type	Cohort (n)	Age (years)	M:F ratio	BMI (kg/m^2^)	No. of cages placed	Cage level(s)	No. of levels instrumented
Diedrich et al. (2001) [25]	4°	20	53.8	6:14	24.7	1.0	L4-L5, L5-S1	1.0
	Rectangle	20	55.2	8:12	25.4	1.0	L4-L5, L5-S1	1.0
Gödde et al. (2003) [8]	Rectangle	22	61	10:12	-	1.4 ± 0.5	L3-4, L4-5, L5-S1	1.4 ± 0.5
Hong et al. (2017) [7]	15°	67	62.5 ± 12.6	28:35	-	1.5 ± 0.5	L4-5, L5-S1	1.5 ± 0.5
	8°	49	60.9 ± 13.2	15:34	-	1.2 ± 0.4	L4-5, L5-S1	1.2 ± 0.4
	4°	65	61.5 ± 10.8	26:39	-	1.3 ± 0.5	L4-5, L5-S1	1.3 ± 0.5
Yi et al. (2020) [19]	Rectangle^†^	18	57.9 ± 7.0	2:16	23.4 ± 3.5	3.5	-	10.9 ± 3.6
Jagannathan et al. (2009) [12]	Rectangle	80	63.2	22:58	-	1.3 ± 0.5	L1-L2, L2-L3, L3-L4, L4-L5, L5-S1	1.3 ± 0.5
Liang et al. (2020) [26]	Rectangle	98	53.0 ± 7.0	41:57	-	1.0	L2-L3, L3-L4, L4-L5, L5-S1	1.0
Sabou et al. (2019) [27]	8°	64	70.3 ± 8.9	13:51	27.1 ± 4.5	3.3 ± 0.8	L1-L2, L2-L3, L3-L4, L4-L5, L5-S1	6.3 ± 2.6

The expandable cages yielded similar radiographic outcomes as 15° and 8° static cages (Table 4). Hong et al. [7] reported a mean increase in lumbar lordosis of 11.8° with the 15° cage, which was similar to the 10.5° ± 14.8° increase observed with expandable cages. While Hong et al. only produced a 6.4° improvement in lumbar lordosis with 8° cages, Sabou et al. [27] observed a 13.3° increase in lumbar lordosis with the same cages, which did not significantly differ from the observed increase with expandable cages (p = 0.34). Expandable cages consistently decreased SVA by a greater magnitude than either 15° or 8° cages, but the change in SVA observed by Sabou et al. with 8° cages was nonsignificant (p = 0.33).

**Table 4:** Lumbar lordosis and sagittal vertical axis by static cage type from spine deformity cohorts reported in the literature. When available, values are reported as mean ± standard deviation. “Δ” represents the change in the radiographic measure after the index operation. P-values represent two-tailed t-tests comparing the postoperative change in lumbar lordosis/sagittal vertical axis of the identified study and that of the expandable cage cohort. Significance is set at p ≤ 0.05.

Type of cage	Study	Lumbar lordosis				Sagittal vertical axis (mm)			
		Pre-Op	Post-Op	Δ	P	Pre-Op	Post-Op	Δ	P
15°	Hong et al. (2017) [7]	31.1	42.9	11.8	-	48.6	31.7	−16.9	-
8°	Hong et al. (2017) [7]	32.7	39.1	6.4	-	50.1	36.1	−14.0	-
	Sabou et al. (2019) [27]	28.1 ± 18.2	41.4 ± 10.9	13.3 ± 21.2	0.34	60.8 ± 56.8	45.9 ± 41.5	−14.9 ± 55.3	0.33
4°	Diedrich et al. (2001) [25]	58.5 ± 14.0	55.1 ± 13.3	−3.4 ± 19.3	0.0003	-	-	-	-
	Hong et al. (2017) [7]	35.8	41.5	5.7	-	47.2	35.7	−11.5	-
Rectangle	Diedrich et al. (2001) [25]	58.2 ± 12.4	52.4 ± 12.1	−5.8 ± 17.3	0.0004	-	-	-	-
	Gödde et al. (2003) [8]	55.0	48.0	−7.0	-	-	-	-	-
	Yi et al. (2020) [19]	37.4 ± 12.2	42.1 ± 9.3	4.7 ± 15.3	0.22	-	-	-	-
	Jagannathan et al. (2009) [12]	36.0 ± 4.5	55.1 ± 6.6	19.1 ± 8.0	<0.0001	-	-	-	-
	Liang et al. (2020) [26]	37.7 ± 12.0	43.0 ± 9.5	5.3 ± 15.3	0.10	-	-	-	-

On balance, expandable cages improved lumbar lordosis compared to both 4° and rectangular static cages. Studies utilizing 4° cages reported a significantly lower mean decrease in lumbar lordosis of 3.4° compared to expandable cages (p < 0.0001), or an increase of only 5.7°, compared to larger increases observed with expandable cages. The use of expandable cages resulted in a greater correction of lumbar lordosis for all but one cohort utilizing rectangular cages. The changes in lumbar lordosis reported by Diedrich et al. [25] were significantly lower than that observed in the expandable cage cohort (p = 0.0004). Jagannathan et al. [12] was the only study employing rectangular cages that reported a significantly greater increase in lumbar lordosis than was achieved with expandable cages (p < 0.0001).

Previous work by Hong et al. [7] and Gödde et al. [8] has shown that, up to a point, increasing the lordosis of the static interbody cage increases both segmental and lumbar lordosis. While most expandable cages lack baseline lordosis, a hallmark of the expandable cage’s design is its ability to increase disc height at the implanted levels [28]. The authors hypothesized that a hyperlordotic expandable interbody cage increases disk height through expansion of the interbody cage, which could yield additional benefits in terms of lumbar lordosis and sagittal balance while obviating the need to conduct an osteotomy. Expandable cages yielded superior improvements in lumbar lordosis in all but one study of rectangular and 4° static cages [7,8,12,19,25,26]. While Jagannathan et al. [12] yielded greater improvements in lumbar lordosis using rectangular cages than was observed in this expandable cage cohort (19.1 ± 8.0 vs. 9.8 ± 14.5, p < 0.0001), these patients had a lesser degree of preoperative lumbar lordosis (36.0 ± 4.5 vs. 33.1 ± 17.2). Further, both Diedrich et al. [25] and Gödde et al. [8] reported decreases in lordosis with the use of static 4° and rectangular cages, respectively, with the remaining cohorts reporting modest gains in lumbar lordosis of 4.7-5.7°. Expandable cages led to similar or better improvements in lumbar lordosis compared to 8° and 15° static cages [7,27]. Hong et al. [7] reported a mean postoperative increase in lumbar lordosis of 11.8° and 6.4° for 15° and 8° cages, respectively. While the mean increase in postoperative lumbar lordosis of 10.5° observed with expandable cages outperformed Hong et al.’s 8° cage cohort, there was no significant improvement in lordosis compared to Sabou et al.’s [27] study of patients treated with static 8° cages (p = 0.34). Additionally, the use of expandable cages resulted in a greater decrease in SVA than was observed in any study of static cages [7,27]. While these differences were nonsignificant, expandable cages should be considered in patients requiring a greater degree of SVA correction. These results suggest that, at worst, expandable interbody cages are equivalent to static cages in open spinal fusion. Especially for rectangular and 4° cages, there may be a significant benefit to radiographic outcomes with the use of expandable cages. Further, the lumbar lordosis correction observed with expandable cages in this cohort was in the notable absence of any osteotomies. The addition of this technique could further increase correction in these patients. Given the lower complication rates and subsistence rate observed with expandable cages, they should be considered a viable alternative to static cages in open deformity surgery [10,28].

Commonly cited indications for higher-order osteotomies such as the pedicle subtraction osteotomy (PSO) include the need for lumbar lordosis correction >25° and a sagittal imbalance of greater than 100 mm [29]. While highly effective, PSOs come with an increased risk of intraoperative complications, especially dural tears and nerve root injuries [30]. Six patients in this study did experience a correction in lumbar lordosis of at least 25° and four patients experienced an improvement in sagittal balance by greater than 100 mm. This suggests that even in those patients necessitating a greater correction, there may exist a subgroup of patients in which expandable cages, if placed properly, could be a viable alternative to the more invasive PSO. Given the decreased morbidity associated with expandable cage placement, more work is needed to characterize this subpopulation in which expandable cages may yield a similar degree of correction.

This study is limited by the fact that it is a single cohort of 45 patients from a single institution, which may introduce bias. While all pre- and postoperative imaging must have occurred within a year of surgery, individual patients had imaging completed at different intervals, which makes for imperfect comparisons. For a minority of cohorts identified by literature search, no SD for the change in radiographic parameters was reported. The methods used to impute these SDs, while grounded in the literature, may also introduce bias. This study compared the clinical and radiographic outcomes of patients treated with expandable cages to those of cohorts treated with static cages in the literature. Given that cohorts were treated by different surgeons at different centers using marginally different numbers of interbody cages, these comparisons will always be imperfect. A multi-institutional randomized controlled trial would help compare expandable cages to the different varieties of static cages to ascertain the true differences in outcomes.

## Conclusions

Expandable cages are a safe and effective means of restoring lumbar lordosis and sagittal imbalance in open spinal deformity surgery, with outcomes that are comparable to those reported for other types of cages in the literature. These cages yield greater improvements in lumbar lordosis and sagittal imbalance than rectangular and 4° lordotic static cages while reducing intraoperative complications compared to rates reported in the literature. Our study suggests that there may be a subset of spine deformity patients who could realize a similar degree of lordosis and sagittal correction that is achieved with PSO by using expandable interbody cages instead.
